# Supplementary material for: Higher-order interactions shape collective dynamics differently in hypergraphs and simplicial complexes
Source: Nat Commun. 2023 Mar 23;14:1605. doi: 10.1038/s41467-023-37190-9 (PMC10036330; doi:10.1038/s41467-023-37190-9)
Supplement: Supplementary file 1 — Supplementary Information [file 41467_2023_37190_MOESM1_ESM.pdf]

Supplementary Information

Higher-order interactions shape collective dynamics differently in hypergraphs and simplicial complexes

Yuanzhao Zhang *[1], Maxime Lucas *[2,3], and Federico Battiston[4]

[1]*Santa Fe Institute, 1399 Hyde Park Road, Santa Fe, NM 87501, USA*
[2]*ISI Foundation, Via Chisola 5, 10126 Torino, Italy*
[3]*CENTAI Institute, 10138 Torino, Italy*
[4]*Department of Network and Data Science, Central European University, 1100 Vienna, Austria*

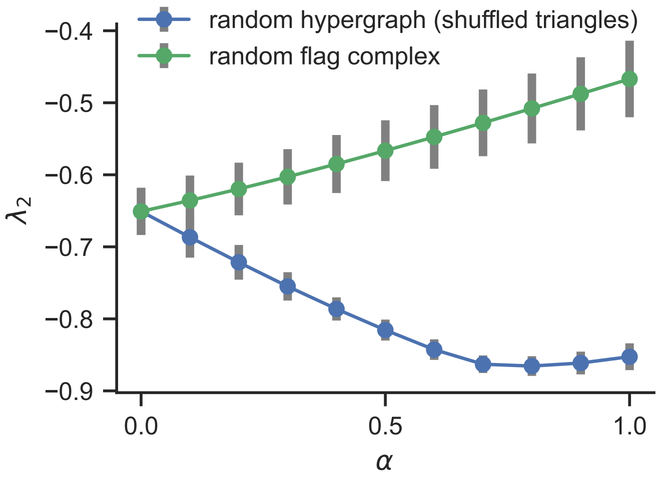

Supplementary Figure S1: **Synchronization is enhanced by higher-order interactions in random hypergraphs but is impeded in simplicial complexes.** Here, we build the simplicial complexes as random flag complexes by generating ER graphs with wiring probability p and filling all closed triangles with 2-simplices. For each realisation, we build a corresponding random hypergraph by keeping the same 1-hyperedges but move each 2-hyperedge to a random location. This ensures that the random hypergraph has the same number of 1-hyperedges and 2-hyperedges as the original simplicial complex. The maximum transverse Lyapunov exponent λ_2 is plotted against α for random hypergraphs (blue) and simplicial complexes (green). As α is increased, the coupling goes from first-order-only ($\alpha = 0$) to second-order-only ($\alpha = 1$). Each point represents the average over 5 independent hypergraphs or simplicial complexes with $n = 100$ nodes. The error bars represent standard deviations. We set $p = 0.4$ for the simplicial complexes.

---

*Y.Z. and M.L. contributed equally to this work.

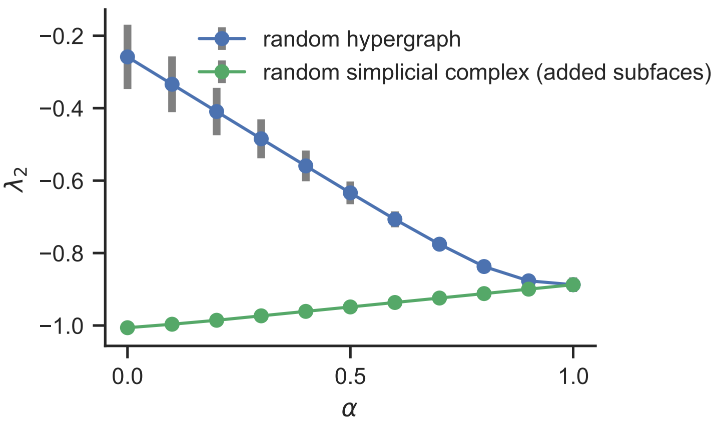

Supplementary Figure S2: **Synchronization is enhanced by higher-order interactions in random hypergraphs but is impeded in simplicial complexes.** Here, we build the random hypergraphs by generating each possible 1-hyperedge and 2-hyperedge with probabilities p and $p_\triangle$, respectively. For each realisation, we build a corresponding random simplicial complex by adding 1-hyperedges to satisfy the inclusion condition. The maximum transverse Lyapunov exponent λ_2 is plotted against α for random hypergraphs (blue) and simplicial complexes (green). As α is increased, the coupling goes from first-order-only ($\alpha = 0$) to second-order-only ($\alpha = 1$). Each point represents the average over 5 independent hypergraphs or simplicial complexes with $n = 100$ nodes. The error bars represent standard deviations. We set $p = p_\triangle = 0.1$ for the random hypergraphs.

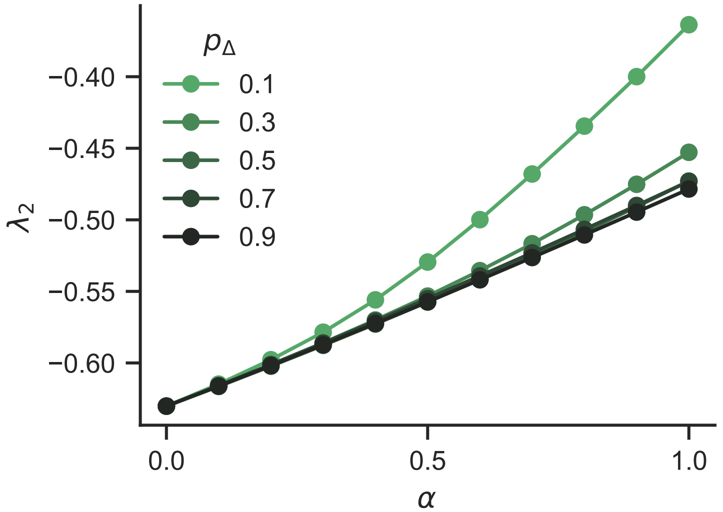

Supplementary Figure S3: **Synchronization is impeded by higher-order interactions in simplicial complexes regardless of the probability of closed triangles being filled.** Here, we build the simplicial complexes as random flag complexes by generating ER graphs with wiring probability p and filling closed triangles with probability $p_\triangle$, for a range of $p_\triangle$ values. The maximum transverse Lyapunov exponent λ_2 is plotted against α. As α is increased, the coupling goes from first-order-only ($\alpha = 0$) to second-order-only ($\alpha = 1$). Each point represents the average over 50 independent simplicial complexes with $n = 100$ nodes. We set $p = 0.4$ for the generation of the original ER graphs.

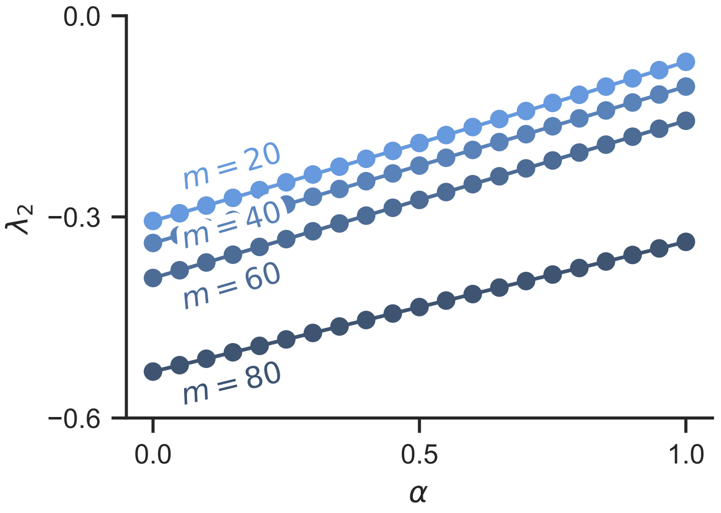

Supplementary Figure S4: **Nonpairwise interactions impede synchronization in simplicial complexes constructed from scale-free networks.** Here, each curve corresponds to a simplicial complex constructed from a scale-free network with $n = 300$ nodes and mean degree $2m$. A scale-free network of n nodes is grown by starting from an m-clique and attaching each new node to m existing nodes. The probability of attachment is proportional to the current degrees of the existing nodes.

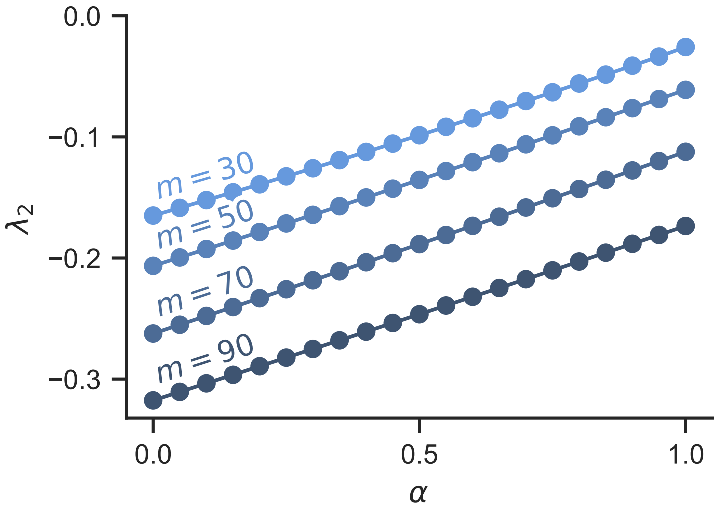

Supplementary Figure S5: **Nonpairwise interactions impede synchronization in simplicial complexes constructed from small-world networks.** Here, each curve corresponds to a simplicial complex constructed from a small-world network with $n = 300$ nodes and mean degree m. A small-world network of n nodes is constructed by starting from a ring network with each node joined with its m nearest neighbors and randomly rewiring each link with probability $p_r = 0.15$.

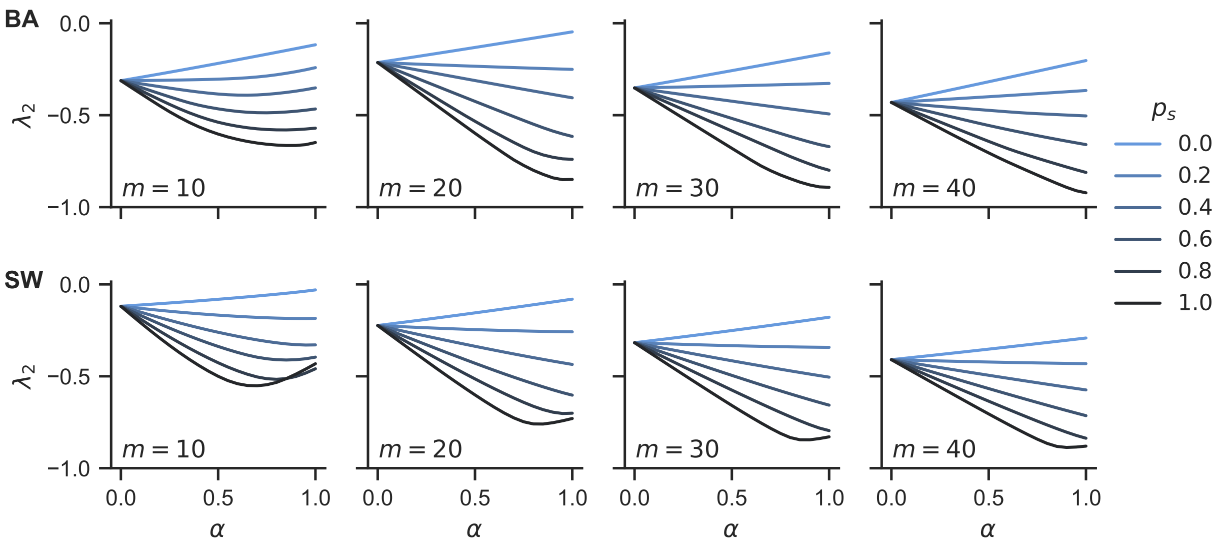

Supplementary Figure S6: **Synchronization stability of hypergraphs constructed from synthetic networks.** Analog of main text Fig. 5, but for scale-free (BA) networks and small-world (SW) networks. The hypergraphs are constructed in the same way as in main text Fig. 5. For each value of p_s, we plot synchronization stability λ_2 (averaged over 100 independent realizations) as a function of the control parameter α. We fix the network size to $n = 100$ for both classes of networks. The BA networks have a mean degree of $2m$, whereas the SW networks have a mean degree of m. Regardless of the mean degree, the role of higher-order interactions always transitions from impeding synchronization to promoting synchronization as p_s is increased.

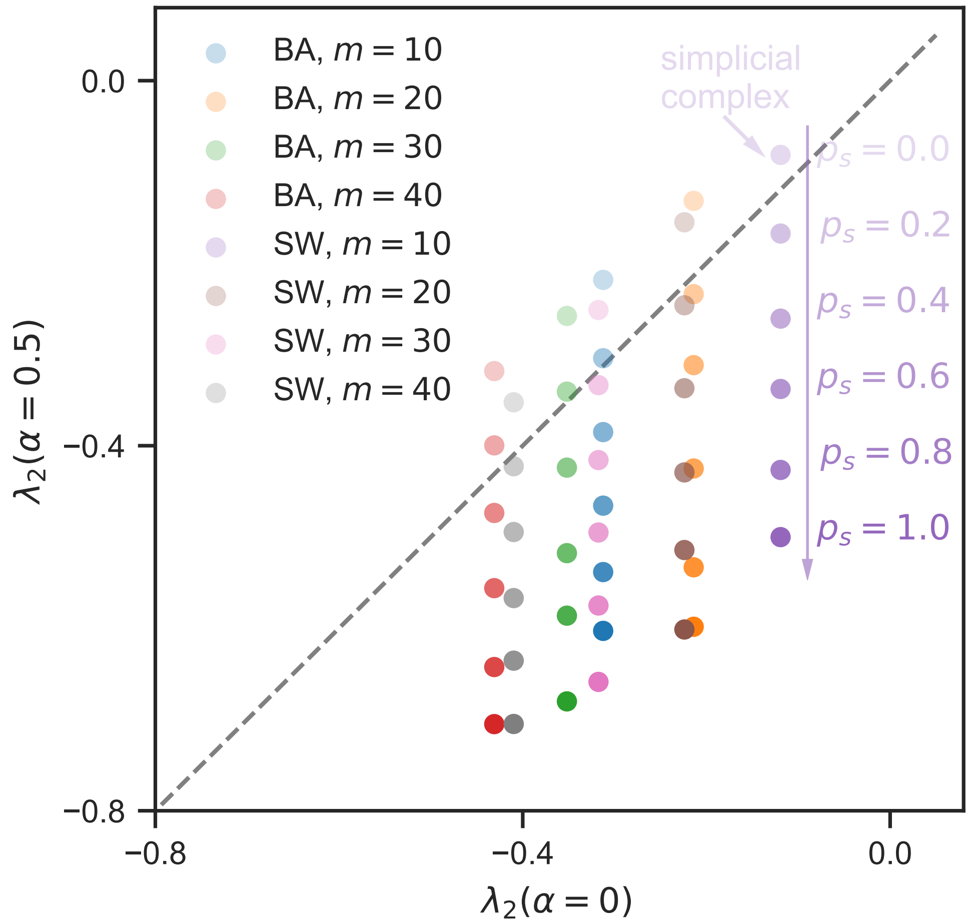

Supplementary Figure S7: **Nonpairwise interactions enhance synchronization in hypergraphs constructed from scale-free (BA) networks and small-world (SW) networks, except when the hypergraph structure is close to being a simplicial complex.** This plot uses the same data as in Supplementary Fig. S6 and is the analogy of main text Fig. 6 for synthetic networks.

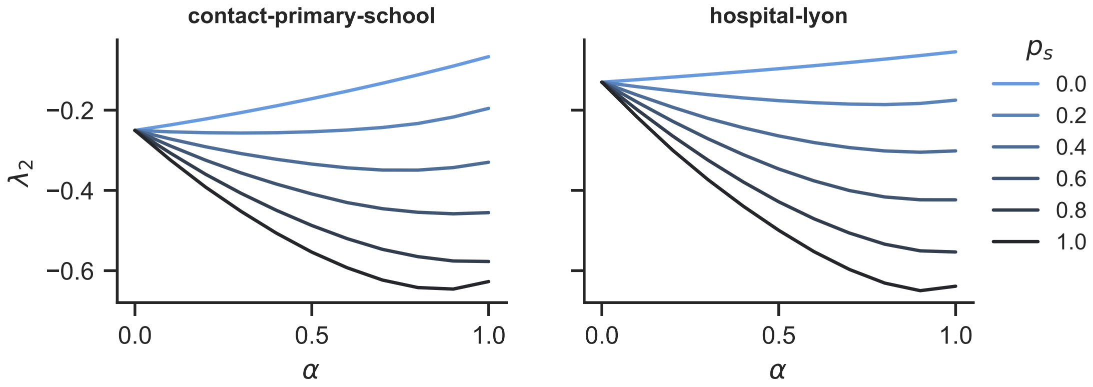

Supplementary Figure S8: **Analog of main text Fig. 5 based on real-world hypergraphs.** The hypergraphs come from social contacts in a Lyon hospital and a primary school. They are known to be close to being simplicial complexes [1]. From these datasets, we keep hyperedges up to order two and consider the largest connected component. For each value of p_s, we plot synchronization stability λ_2 (averaged over 100 independent realizations) as a function of the control parameter α. The role of higher-order interactions quickly transitions from impeding synchronization to promoting synchronization as p_s is increased.

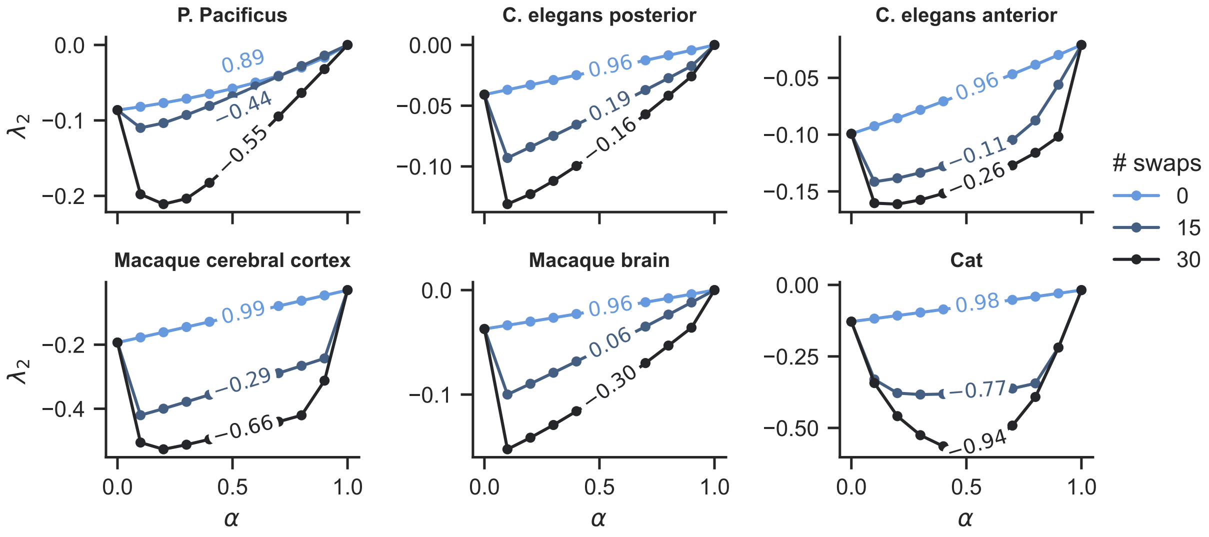

Supplementary Figure S9: **Cross-order degree correlation affects synchronization stability in systems with mixed pairwise and nonpairwise interactions** ($0 < \alpha < 1$)**.** This plot uses the same networks as in main text Fig. 5 and extends main text Fig. 7. Curves are colour-coded by the number of pairs of nodes that were selected to swap their 2-simplices memberships: 0, 15, or 30. The resulting degree correlation is indicated on each curve.

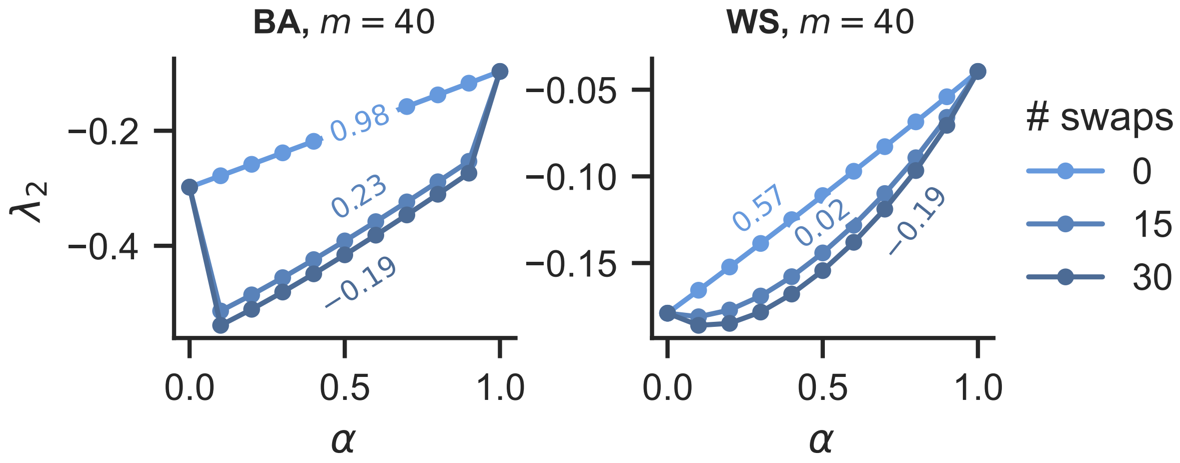

Supplementary Figure S10: **Cross-order degree correlation affects synchronization stability in systems with mixed pairwise and nonpairwise interactions** ($0 < \alpha < 1$)**.** The hypergraphs are constructed from scale-free and small-world networks, with the same parameters as in Supplementary Figs. S4 and S5: $n = 300$, $m = 40$, and $p_r = 0.15$. Curves are colour-coded by the number of pairs of nodes that were selected to swap their 2-simplices memberships: 0, 15, or 30. The resulting cross-order degree correlation is indicated on each curve.

References

[1] Lotito, Q. F., Musciotto, F., Montresor, A. & Battiston, F. Higher-order motif analysis in hypergraphs. *Commun. Phys.* **5**, 79 (2022).
